# Opening of the Royal Eye Hospital, Southwark

**Published:** 1893-01-14

**Authors:** 


### NEW HOSPITALS.

### OPENING OF THE ROYAL EYE HOSPITAL, SOUTHWARK.

On Thursday, December the 15th, this hospital, which has been entirely rebuilt, was opened by his Royal Highness the Duke of York. The new buildings are on a much more extensive scale than the original hospital, and are now able to contain forty beds.

The opening ceremony was held in the Surrey Theatre adjoining the hospital, which was lent for the occasion by Mr. George Conquest.

By half-past twelve nearly every seat was occupied. There was a sprinkling of nurses in the audience, amongst whom we noticed Miss Monk, of King's, with a party of her nurses. We also recognised Miss Ellen Terry amongst the occupants of one of the boxes.

Soon after one o'clock the members of the Reception Committee appeared and took the plac s res rved for them. Almost immediately the band of the Royal Marine Artillery, stationed at the back of the stage, played the opening bars of the National Anthem, and the audience rose to their feet as the Duke of York, accompanied by the Duke of Cambridge, entered, and was marshalled to his place. Two or three prayers were said by the Bishop of Rochester, producing a certain sense of the incongruous when the surroundings were taken into consideration, and then the Duke of Cambridge, as patron of the hospital, after speaking in praise of the way arrangements had been carried out in the new building, welcomed the Duke of York in the name of the Committee on his visit to South London.

A gold key was handed by the architect to His Royal Highness the Duke of York, who fitted it into the model of a lock placed on the table before him. This performance, to quote the programme, was to complete the electric circuit, open the hospital, illuminate the hall for a photograph, and discharge the signal cannon, whereupon the bands played "God Bless the Prince of Wales."

This accomplished, the Duke in a speech short and to the point, declared the hospital open, and having signed the roll recording the ceremony, duly received the thanks of the Chairman, the official part of the proceedings ended, and the two Royal visitors departed.

More band playing, and an "oxyhydrogen lantern demonstration of special features in the new hospital," followed.

This hospital claims to be a "model building" "unsurpassed in the world," but here we venture to suggest that however well the scientific department has been dealt with, in some respects the comfort of both nurses and patients seems to have been rather left out of the question. One notable instance of this is the lack of washing conveniences. One bath on admission is apparently to suffice for the patients, as there is none on the same floors as the wards, except a shower-bath, and no fixed basins. There is also but one very small bath, which is to be shared between nurses and servants. The arrangements for the nurses appear to us altogether to leave a good deal to be desired, both as to the inadequate bath-room, and also in small matters, such as the jugs and basins provided for use in their own sleeping-rooms, the unbreakable jug being very small and of a different colour and pattern to the basins which accompany them.

That the nurses' dining-room should also be the servants' sitting and meal room is a curious arrangement, but it is not worse than the one which provides no day-room for the patients at all.

The bedsteads by Longford Company at Warrington are made on an excellent plan; the head part can be removed and replaced by small operation tables, which fit into the corner posts, made hollow for the purpose. The general wards contain seven and eight beds each, and there are various small private wards for paying patients. The whole building is fireproof, and is heated throughout by hot water. The nurses' rooms are on the top floor, and the roof is flat and provided with garden seats, with the view of being used as a recreation ground. A convenient plan has been followed everywhere in the building, namely, that of rounding all corners, thereby preventing the accumulation of dust, and allowing any blind inmates of the hospital to find their way about without danger of coming in contact with sharp projections. With the same idea the doors are made to pull and push, not to fasten in the ordinary way. Altogether the hospital needs but a few alterations or additions to be considered a satisfactory institution.

